# Gratuitous Medical Services to the Public

**Published:** 1882-03

**Authors:** 


### Editorial.

### GRATUITOUS MEDICAL SERVICES TO THE PUBLIC.

The *Buffalo Medical and Surgical Journal,* in the course of a recent editorial, wherein the management of the sanitary affairs of that city is freely discussed, says: "If the question were presented what suggestions have we to offer in the organization of such a board, we should not hesitate to express our conviction that a board of health should be composed of at least five members, of which three should be medical men, to serve without compensation."

It is the suggestion embodied in the last clause of this paragraph which merits thoughtful consideration, and which we propose to make the basis of a few brief observations; for, we believe that it represents one of the gravest maladies now affecting the body medical.

Why should the physician render such services gratuitously? We frankly confess that not a single argument in favor of this course presents itself, while a great many good and sufficient reasons why they decidedly should not readily occur to us.

The general public is not an object of charity. It is, or ought to be, able to pay for what it receives, and, if it stands in need of advice from medical men, it should be willing to offer fair compensation. If the advice is worth nothing it should not be asked or accepted by the public, or, if the necessity for skilled counsel does not exist, the advice may, with entire propriety, be withheld; but if it is of value to the community or the state, a *quid pro quo* should be demanded and should be given. The medical profession surely is not pre-eminently in need of sanitary observances on the part of the public. As a class, it is, perhaps, less dependent upon such safeguards than most other people, for the peculiar knowledge which it possesses, and the position which it occupies in the community, generally enable it to protect itself from outside sources of disease ; nay more, a very narrow and very selfish view of the question would seem to sustain the opinion that public sanitation is inimical to medical interests. We certainly do not indorse this sentiment. But be this as it may, it is a fact highly creditable to the medical profession that self-interest does not furnish the motive which places it always in the lead on questions of public sanitary improvement; rather is it the recognition of the necessity for attention to these matters, which it, better than any other class of citizens, can justly appreciate. This activity for the public safety, we repeat, is praiseworthy, but no good can come from carrying it too far. It is subject to abuse, and, in our deliberate opinion, it is abused.

Every physician knows from personal experience that the most exacting, unreasonable and ungrateful patients are those who pay no fees, and that, as a rule, services for which a regular fee is demanded and paid, are the most highly appreciated. What is true of the individual is true also of the aggregation of individuals known as the public. Free service is lightly esteemed, but services that are liberally compensated—whether it be that they are valued because they are paid for, or paid for because they are valued, we will not here undertake to determine—but the mere fact of compensation, we say, increases their importance in the public estimation and gives to them a far wider and more influential power.

The profession is not entirely free from blame in this matter, and while we recognize the fact that the errors, as they appear to us, are the result of a laudable desire to benefit the people, we nevertheless, cannot regard such gratuities otherwise than as a serious mistake, and unjust alike to the government—be it federal, state or municipal—and to the medical profession itself.

This is not one of the necessary evils that must be endured, for physicians have a remedy within their reach, and it is the failure to utilize the remedy which makes them responsible. Let them refuse to serve the public gratuitously, and the public will soon recognize the necessity for their services, and will as surely willingly offer a just return for what it receives. The public, moreover, will be quick to arrive at the conclusion that those persons who are anxious to render gratuitous service are not fit to serve it at all.

It is not indicative of a want of philanthropy, of public spirit or of charity to pursue the course indicated. It is only right. Right in every view of the case and equitable toward the public and toward the profession.

We respectfully commend these suggestions to our cotemporary in the commendable effort it is making to secure an improved sanitary organization for its prosperous city.

